# Fertility desire and associated factors among people living with HIV attending antiretroviral therapy clinic in Ethiopia

**DOI:** 10.1186/s12884-014-0382-2

**Published:** 2014-11-20

**Authors:** Dereje Bayissa Demissie, Bosena Tebeje, Temamen Tesfaye

**Affiliations:** Department of Nursing and Midwifery, College of Medicine and Health Science, Ambo University, Ambo, Ethiopia; Department of Nursing and Midwifery, College of Public Health and Medical Sciences, Jimma University, Jimma, Ethiopia

**Keywords:** People living with HIV, Ethiopia, Fertility desires

## Abstract

**Background:**

The reproductive decisions made by PLHIV and their partners have a long-term consequences for the survival and wellbeing of their families and a society at large. Evidence relating to fertility and reproductive intentions among PLHIV is rare, despite the fact that more than 80% of PLHIV are of reproductive age. The aim of the study was to determine fertility desire and associated factors among PLHIV attending ART clinic in Fitche Hospital.

**Methods:**

A facility based cross-sectional study design with both quantitative and qualitative data collection methods was employed from February21-April 20th, 2013. The study participants were selected by using simple random sampling technique. A pre- tested structured questionnaire was used to collect data. Both bivariate and multivariate logistic regressions were used to identify associated factors.

**Result:**

The prevalence of fertility desire of PLHIV in Fitche Hospital was 133(39.1%) with 95% CI of (34.3% -44.3%). This study identified that factors found to be associated with fertility desire were: − Age from 18-29y [AOR = 3.95, 95% CI: 1.69 - 9.22) and 30-39y (AOR = 3.91, 95% CI: 1.90 -8.19)], marital length ≤4y [AOR = 5.49, 95% CI: 2.08-14.51), within 5-9y (AOR = 4.80, 95% CI: 2.14-10.78) and 10-14y (AOR = 2.82, 95% CI: 1.19 -6.63], had not biological living children [AOR = 11.42, 95% CI: 3.27-39.90) and had more than one child (AOR = 3.67, 95% CI: 1.27-10.62)], community pressure [AOR = 3.67, 95% CI: 1.54-8.70], partner fertility [AOR = 7.18, 95% CI: 3.39-15.22)], duration HIV diagnosis≤1y[AOR = 4.99, 95% CI: 1.91-13.09], disclosed HIV serostatus [AOR = 3.9, 95% CI: 1.37-11.10] and partner sero-difference [AOR = 2.05, 95% CI: 1.01- 4.15] were some of the factors significantly associated with fertility desire.

**Conclusion:**

The prevalence of fertility desire of PLHIV in the study area was 39.1%. In this study:- age, marital length, biological child, partner, community pressure, duration of HIV-diagnosis, discordant HIV-test and disclosure of HIV-serostatus to partner were demonstrated to have more associations with fertility desire among PLHIV, therefore, these factors should be emphatically considered during PLHIV’s reproductive health program development.

## Background

Human immuno virus (HIV), is the virus that causes acquired immunodeficiency syndrome (AIDS), has become one of the world’s most serious health and development challenges [1]. The first case was reported in 1981 and currently, more than 30 years later, there are approximately 34 million people living with HIV and nearly 30million people have died by AIDS-related causes since the beginning of the epidemic [1,2]. Ethiopia is among the countries that are on top of a list of nations hard hit by HIV pandemic. According to Ethiopia Demographic Health Survey reported in 2011, the prevalence of HIV among reproductive age (15–49 years) were 1.5% and the prevalence of HIV among women were 1.9% whereas in men 1% [3].

Fertility desires is intention to have more children despite the diagnosis of HIV, whereas intentions denote a commitment to implementing fertility desires. Therefore, intention comprises both desire and planning aspects. Desire for a child means that expression of PLHIV to have child in the future, despite the diagnosis of HIV [4].

Historically, policies in many countries discouraged HIV-infected individuals from having children in order to reduce the number of children born with HIV or born to HIV-infected parents, but a more flexible approach towards reproductive choices of PLHIV has now emerged. This shift has been mainly informed by a reproductive rights approach and universal access to PMTCT/ART interventions and the availability of assisted reproductive techniques for HIV infected people in developed countries which have dramatically reduced the chances of sexual and perinatal HIV transmission [5,6]. This has given rise to the growing recognition of the rights of PLHIV to have children or prevent unintended pregnancies [7]. It is important to study fertility desires and associated factors among PLHIV because HIV can be transmitted in the same way that pregnancy is achieved, that is, through unprotected hetero-sexual intercourse [8].

Thus, unprotected sex among PLHIV, in order to conceive, carries the risk of transmitting HIV to sexual partners and subsequently to children during birth or breast feeding. The reproductive decisions made by PLHIV and their partners have long-term consequences for the survival and wellbeing of their families and society at large [9]. The desire and intent to have children among HIV infected individuals may increase because of improved quality of life and survival following commencement of anti-retroviral treatment and reproductive health service as well [5].

The complex relationship between fertility and HIV/AIDs threatens the preventive strategies against the HIV epidemic in countries like Ethiopia, where the fertility rate is still high and PMTCT utilization low [10]. While the Government of Ethiopia’s HIV policy is supportive of HIV and reproductive health services integration, these services remain predominantly vertical in terms of program administration, funding and service delivery [11]. Reproducing ("giving life") for HIV-positive individuals means transcending the death that appears near, and these figures may be much higher in low-resource settings, where the disease prognosis is still very poor [3]. PLHIV, just like the general population, desire to have children after learning of their HIV-status [12-14]. Unlike the general population, people who know they are HIV infected have additional issues to consider, including potential health risks for (re)infections, vertical transmission of HIV and orphaning. Despite these concerns, studies show that some PLHIV still wish to have children for a range of reasons [5,15]. Most couples living with HIV are of child bearing age and facing difficulty in their choice concerning sexuality and child bearing [13].

Most recently, fertility issues in PLHIV are becoming increasingly important [4]. In a setting with high HIV prevalence and high fertility rates, addressing fertility issues of PLHIV is crucial. However, understanding of the factors associated with fertility desires of PLHIV in Ethiopia is remarkably low. In 2009, only 8 percent of HIV-positive pregnant women received ARV prophylaxis [11]. Evidence relating to fertility and reproductive intentions among PLHIV is rare [16], despite the fact that more than 80% of PLHIV are of reproductive age [7]. HIV positive individuals may or may not have desire to have children and want to use family planning. Hence this study was conducted to identify fertility desires and associated factors among PLHIV attending ART clinics of Fitche Hospital.

## Methods

### Study setting

The study was conducted from February 21st to April 20th of 2013, in Fitche Hospital ART clinic which is found in Fitche town, North shoa Zone, Oromiya Regional State in Ethiopia and 115 Kilo meters North of Capital city of Addis Ababa. According to the national population and housing census of 2007/08 of Ethiopia, the projected population of the zone for 2013 was estimated to be 1,388,617 and from those 6, 951, 87(50.06%) were males. The zone has 2 hospitals, 48 health centers and 268 functioning health posts with estimated potential health service coverage of 91.6%. The total numbers Reproductive ages on HAART and Pre-ART in north shoa zone was 8821.

Fitche Hospital provides different services like Outpatient department, Inpatient department, Maternal Neonatal Child Health and Pre-ART and ART services by different disciplines. The total numbers of reproductive age PLHIV in Fitche Hospital was 2131 (from those 1211 on ART and 920 Pre- ART respectively).

### Study design

A facility based cross-sectional study design with both quantitative and qualitative data collection methods was conducted in ART clinic of Fitche Hospital. The study inclusion criteria were having attended for at least three months, availability of HIV sero-status results, HIV positive diagnosis and reproductive age, 18 to 49 years of age for women and 18 years and above for men.

#### Sampling procedure and sample size determination

For the quantitative study sample size was determined by using single population proportion formula by considering 50% proportion of fertility desire among PLHIV with 95% confidence interval and 5% marginal error. Since the total numbers of patients enrolled to ART clinic at Fitche Hospital were 2131, Population correction formula was used. By considering 10% non-response rate, the final sample size was 357.

A list of all women of reproductive age (18–49 years), and 18 years and above for men who are living with HIV were selected and entered into computer SPSS window 16.0 version from HIMS data base. Computer generated simple random sampling technique was employed to select study respondents by using their Pre-ART card number. During the one -month study period, 340 PLHIVs were recruited into the study by randomly selection.

For qualitative study; all mother support group (four from four mothers) and all peer educators (six from six peer educators) were recruited purposively based on their duration of follow up greater than 10 years and they are expert patient of PLHIV who are working in the hospital.

### Data collection procedures

Data were collected by face to face interview by using structured, pre-tested Amharic and Afan Oromo version questionnaire. The questionnaires were initially prepared in English and translated to Afan Oromo and Amharic and back to English by language experts and researchers to keep the consistency of the questionnaires. Two well-trained diploma nurses who are working in the ART clinic had collected data and one BSC Nurse had supervised during data collection period. Data collectors had cross checked Pre-ART card numbers of all clients who came to ART clinic with sampled card numbers daily.

The filled questionnaires were checked for consistencies and completeness daily by supervisor and principal investigators on the spot. Pre-test of the questionnaire were done on 5% of the sample of PLHIV in Kuyu Hospital which is nearby to Fitche town, to identify any ambiguity, consistency and acceptability of questionnaire, and then necessary corrections were made before the actual data collection.

Qualitative data was collected by ART focal person through in-depth interview by using semi-structured interview guide and conducted in separate room. Voice recorder and field-notes were used to capture the information obtained from the in-depth interview.

### Data processing and analysis

After data collection, each questionnaire was checked for completeness and code was given before data entry. Data was entered, sorted, edited and cleaned for missed values. Data were analyzed by using SPSS version 16.0 statistical packages and presented by frequencies and percentages for categorical variables and means and standard deviations for numerical variables. Bivariate analysis was conducted primarily to check the variables which had an association with the dependent variable individually. Variables associated with the dependent variables at p value <0.2 were then entered in to multiple logistic regression for controlling the possible effect of confounders and finally the variables which had significant association with fertility desire were identified on the basis of adjusted odds ratios (AOR), with 95% CI and p-value (<0.05) to fit into the final regression model. The results were presented using tables, figures and narratives.

### Qualitative data

To add an in-depth of information on fertility desires that could not be captured by quantitative methods alone. Data captured using tape records was translated word by word into English language and color coded, organized and summarized manually under the main thematic area and presented the result by extracted concepts from main themes.

### Operational definition

#### Fertility desires

A psychological state in which someone has the personal motivation to have a child. Those who have motivation to have more children in the future have fertility desire (have fertility desire). Those who have no motivation to have more children have no fertility desire (have no fertility desire).

### Ethical consideration

Ethical clearance letter was initially obtained from Jimma University College of Public Health and Medical Sciences Ethical Committee. Then written consent was secured from Fitche hospital and permission was secured. Verbal informed consent for participation and audio recording of the discussions was obtained from each participant and the collected data were stored in a file, without the name of study participant and password protection of soft copy data and use of key and lock for hard copy data was employed to guarantee confidentiality.

## Result

### Socio demographic characteristics

Of 357 sampled PLHIVs, data were collected from 340 which give a response rate of 95.2%. Among study participants majority 214(62.9%) were females, 144(42.4%) were between the age 30–39 years and range from 18–70 years for males and 18–49 years for females with a mean age of 36.2 ± 9.2 years. Concerning ethnicity, majority of the respondents 265(77.9%) were Oromo and 325(95.6%) of the respondents were Orthodox in religion. With regard to educational status, 132(38.8%) were illiterate and 184(54.1%) were attended primary to secondary school. Concerning occupational status 78(22.9%) were daily labour and family monthly income distribution of respondents, 94(27.6%) had an income less than equal to ≤350 birr per month with average monthly income was 735.9 ± 631.48 standard deviation Ethiopian birr (1USD =18.42 Birr) (Table 1).

**Table 1:** **Sociodemographic characteristics of 340 PLWHA attending ART clinic in Fitche Hospital, Ethiopia, 2013G.c**

**Socio-demographic variables**	**Categories**	**Frequency(n)**	**Percent (%)**
Sex (n = 340)	Male	126	37.1
	Female	214	62.9
Age(n = 340)	18-29 years	85	25.0
	30-39years	144	42.4
	> 40+ years	111	32.6
Ethnicity (n = 340)	Oromo	265	77.9
	Amhara	73	21.5
	Others	2	0.6
Religion (n = 340)	Orthodox	325	95.6
	Protestant	13	3.8
	Muslim	2	0.6
Occupation (n =340)	Daily labor	78	22.9
	Merchant	77	22.6
	Gov’t employed	43	12.6
	House wife	65	19.1
	Farmer	57	16.8
	Unemployment	20	5.9
Level of school (n =340)	Illiterate	132	38.8
	Primary and secondary (1–8)	118	34.7
	High school and preparatory school(9–12)	66	19.4
	College and above	24	7.1
Marital status (n = 340)	Married	195	79.1
	Single	19	8.8
	Windowed	56	12.1
	Divorced/separated	70	20.5
Family Income (n =340)	<=350 birr	94	27.6
	351-500 birr	81	23.8
	501-999 birr	105	30.9
	> = 1000 birr	60	17.6
Residence (n = 340)	Urban area	271	79.7
	Rural area	69	20.3

Key: others (Tigrie & Agnuwak).

### Sexual activity and contraceptive use information of PLHIV

The majority of respondents 234(68.8%) were sexually active. Of which 203(86.76%) had sex with regular partner (husband/wife) and 31(13.24%) were had multiple sexual partners. From the total study participants about 87(25.6%) of them were changed their sexual partner since HIV diagnosis.

The majority of them 169(60.8%) were used condom, of which 120(71.0%) used always. Eighty one (48.0%) of them used dual methods of contraceptive by themselves or their partners of which majority, 73(90.1%) were used Depo-Provera in addition to condom. The main reason mentioned for use of condom, 115(33.8%) were reported that for dual protection (pregnancy/STI/HIV), 35(10.3%) to protect a negative partner, 15(4.4%) fear of re-infection with new stain of HIV and 4(1.2%) advised by health professionals. Whereas for those not used condom the main reasons mentioned were partner objection, feeling it was not comfortable and desired to conceived which account 59(17.4%), 25(7.4%) & 24(7.1%) respectively.

Table 2 shows that the majority 290(85.3%) had living children. From them 59.4% had 1 - 3children and 25.9% had more than 4 children whereas only 50(14.7%) had no biological living children. From those who had biological children 24(8.3%) were previously died related to HIV/AIDs and/or other diseases after learnt their or their partners’ serostatus.

**Table 2:** **Reproductive history and Fertility desire of PLHIV attending ART clinic in Ethiopia 2013**

**Variables**	**Frequency(n)**	**Percent (%)**
Number of living children	n = 340)	
No living child	50	14.7
1 to 3 children	202	59.4
≥ 4 children	88	25.9
Child died related HIV before	n = 290	
Yes	24	8.3
No	266	91.7
Pregnant or partner pregnancy since HIV-Dx	n = 340	
Yes	83	24.4
No	257	75.6
Did you have fertility desire	N = 340	
Yes	133	39.1
No	207	60.9
When do you desire to have a child?	n = 133	
Within next 12 months	24	18.0
Within one to three years	18	13.5
After three years	33	24.8
Not decided when to have a child	58	43.6
Take action to become pregnant/your partner’s?	n = 340	
Yes	35	10.3%
No	305	89.7%
What kinds of action did you take?	35	
stop take contraceptive methods	17	48.6%
discussed fertility intentions with caregivers	11	31.4%)
approach to their partner	7	20.0%
Number of children desire to have in the future?	n = 133	
One child	77	57.9
≥ 2 children	56	42.1
Reason for their current fertility desire	n = 133	
Want at least one child	49	36.8
I did not have desired number	51	38.3
To strengthen marriage	4	3.0
Perceived efficacy of ART/PMTCT	27	20.3
To replace died baby before	2	1.5
Preference of sex for future fertility desire?	n = 133	
Male	41	30.8
Female	23	17.3
No preference(God knows)	69	51.9
Desire of a partner to have child?	n = 203	
Yes	104	51.2
No	99	48.8
Disscuss fertility intention with health profession	n = 340	
Yes	137	40.3
No	203	59.7
Did you face partners or your family pressure for child birth?	n = 340	
Yes	99	29.1
No	241	70.9
Did you faced community pressure for child birth?	n = 340	
Yes	82	24.1
No	258	74.9

From the total interviewed PLHIV’ about 83(24.4%) had at least one pregnancy by themselves or their partners post-HIV diagnosis of which 62.7% was planned. The outcomes of these pregnancies were 66.3% live birth, 14.5% abortion, 7.25% still birth and 12.0% currently pregnant (during study period). The majority of in-depth interview discussants supported this finding, for instance: − as one 24 years female discussant stated: “I want to give birth because my husband is need child to replace ourselves. So, I have to get pregnant soon while my health is good enough. We gotten PMTCT and ART help from hospital, so I don’t have any fear my child will be free of HIV and currently pregnant”.

Table 2 shows that more than half 104(51.2%) partners had fertility desire. Of total respondents about 99(29.1%) was faced their family’s pressure and 80(23.7%) had community pressure for having children. This finding is supported by majority of in-depth interview discussants, for example: − as one 45 yrs/Male explained: “Bearing children is important as one with no children is forgotten when died. Wealth shall be transferred to children otherwise it is lost. My parents and community will not respect me and my property. One without child is not considered born and the community called them as ‘mule/infertile endwodi’.

### Fertility desire of PLHIV

Out of 340 PLHIV interviewed 133(39.1%) with 95% CI of (34.3% -44.3%) had fertility desire of which 24(18.0%), 18(13.5%), 33(24.8%) and 58(43.6%) were desired to have child within next 12 months, within one to 3 years, after three years and did not decided the time when to have children respectively. The main reasons mentioned for their current fertility desire were 36.8% wanted to replaced themselves, 38.3% did not have desired number of children and 20.3% believed that by using ART/PMTCT to get HIV free baby (perceived efficacy of PMTCT and ART) and others reasons like to strength their marriage and replacing previously died baby. Of the total respondents 35(10.3%) were taken action to been pregnant or their partners. Of which 17(48.6%) were stopped taken contraceptive methods.

From those who have fertility desire about 77(57.9%) and 56(42.1%) will have future desire number of a child, one and more than two children in the future and ranges from 1 to 4 children to achieved their fertility desire respectively. A 30 years woman described: “I have a boy and a girl so I need a brother for son and a sister for my daughter when my health is improved and CD4 is better and after I discussed with health professions. My husband has no biological child before he want child strongly, in the presence of treatment having children is preferable as it is to replace one self and it is natural to do so”, With regarding to the preference of sex of child born in the future about 69(51.9%) were not preferred any sex and 41(30.8%) was preferred male sex.

The main reasons mentioned for their current fertility desire were 36.8% wanted at least one child to replaced themselves, 38.3% did not have desired number of children and 20.3% believed that by using ART/PMTCT to get HIV free baby (perceived efficacy of PMTCT and ART) and others reasons like to strength their marriage and replacing previously died baby. Of the total respondents 35(10.3%) were taken action to been pregnant or their partners of which 17(48.6%) were stopped taken contraceptive methods. Out of the total study participants only 137(40.3%) were discussed about fertility intentions and others reproductive health needs with health professionals during follow up care.

With regarding to PLHIV who have no fertility desire 207(60.9%) were mentioned their main reasons for not having fertility desire; 69(33.3%), 59(28.5%), 34(16.4%) and 45(21.7%) were lack of adequate income, already achieved desired numbers of children, child bearing compromised their or partner’s health and the rest mentioned fear of MTCT and fear of infected their partner while try to conceive respectively. This finding is supported by most of in-depth interview discussants, for instance: − as one 36 years Woman discussant explained: “I have three children, me and the smallest are on ART but my husband is negative. Now I feel very sorry for the suffering of my baby hence I do not repeat the same sin by bearing positive child.”

Table 3 showed that Out of the total study participants 209(61.5%) had good knowledge about prevention of mother-to-child transmission of HIV (PMTCT), of which 136(63.6%) were women and 73(57.9%) were men. On the other hand, with regarding to HIV transmission from mother to child about 298(87.6%) study respondents were knowledgeable and 202(59.4%) have positive attitude towards PMTCT.

**Table 3:** **Disease related factors of PLHIV attending ART clinic in Fitche Hospital Ethiopia, 2013**

**Variables**	**Frequency(n)**	**Percent (%)**
HIV transmits from mother to child?	n = 340	
Yes	298	87.6
No	42	12.4
Time of MTCT?	n = 340	
During pregnancy	224	65.9
During labor	163	47.9
During breast feeding	140	41.2
Have information about PMTCT	n = 340	
Yes	216	63.5
No	124	36.5
Attitude toward PMTCT?	n = 340	
Yes (positive attitude)	202	59.4
No (negative attitude)	138	40.6
Knowledge about PMTCT?	n = 340	
Knowledgeable	209	61.5
Not knowledgeable	131	38.5
Start ART	n = 340	
Yes	310	91.2
No	30	8.8
Partner HIV status?	n = 203	
Positive (concordant)	143	70.4
Negative (discordant)	60	29.6
Your partner started ART if HIV + Ve?	n = 143	
Yes	100	70.0
No	43	30.0
Duration of time since enrolled to ART?	n = 340	
6 months to 1 year	50	14.7
2 to 4 years	130	38.2
≥5 years	160	47.1
Current CD4 count?	n = 340	
≤350 cells/m3	153	45.0
≤350 cells/m3	187	55.0
WHO stage when enrolled to ART?	n = 340	
Stage 1	59	17.4
Stage 2	48	14.1
Stage 3	222	65.3
Stage 4	11	3.2
Disclosed HIV status to partner?	n = 223	
Yes	195	87.4
No	28	12.6
Disclosed HIV serostatus to family?	n = 340	
Yes	254	74.7
No	86	25.3
Difference in fertility desire after started HAART?	n = 310	
Yes	103	33.2
No	207	66.8
Current health status affected future child bearing intention?	n = 340	
Yes	204	60.0
No	136	40.0

### Factors associated with fertility desire among PLHIV

(Table 4 below) factors found significantly predictive of fertility desire for PLHIV to have more children were: − Age from 18–29 years were 3.95 times [AOR, (95% CI), 3.95(1.69 - 9.22) and 30-39 years were 3.91 times (1.90 - 8.20)] more likely to have fertility desire as compared to age 40+ years respectively.

**Table 4:** **The overall fertility desire predictors of PLHIV attending ART clinic in Fitche Hospital, Ethiopia, 2013G.c**

**Variables**	**Fertility desire (n = 340)**		**COR (95% CI)**	**AOR (95% CI)**
	**Yes (%)**	**No (%)**		
Age				
18-29 years	46 (13.5)	39 (11.5)	4.51 (2.41,8.45)***	3.95 (1.69, 9.22)***
30-39 years	64 (18.8)	80 (23.5)	3.06 (1.74, 5.38)***	3.91 (1.90, 8.20)***
> =40 years	23 (6.8)	88 (25.9)	1.00	1.00
Duration stayed with partner/marital length (n = 203)				
≤4 years	24 (11.8)	13 (6.4)	5.333 (2.25,12.63)***	5.49 (2.08, 14.51)***
5-9 years	34 (16.7)	20 (9.9)	2.63 (1.170, 5.89)***	4.80 (2.14, 10.78)***
10-14 years	20 (9.9)	22 (10.8)	2.031 (2.275, 10.60)**	2.82 (1.19, 6.63)**
>15 years	18 (8.9)	52 (25.6)	1.00	1.00
Number of living children				
have no living child	38 (11.2)	12 (3.5)	20.06 (8.24,48.83)***	11.42 (3.27, 39.90)***
have 1 to 3 children	83 (24.4)	119 (35)	4.540 (2.24,9.21)***	3.67 (1.27, 10.615)**
have ≥4 children	12 (3.5)	76 (22.4)	1.00	1.00
Face community pressure for having children				
Yes	61 (18.0)	19 (5.6)	8.29 (4.63,14.85)***	3.67 (1.54, 8.704)***
No	72 (21.3)	186 (55.0)	1.00	1.00
Partner fertility desire				
Yes	78 (22.9)	26 (7.6)	13.04 (6.74,25.25)***	7.18 (3.39, 15.22)***
No	20 (5.9)	79 (23.2)	1.00	1.00
Duration since HIV Dx				
6 months to 1 year	27 (7.9)	23 (6.8)	2.24 (1.176,4.27)**	5.0 (1.91, 13.09)***
2 to 4 years	51 (15.0)	79 (23.2)	1.82 (0.942, 3.51)*	
≥5 years	55 (16.2)	105 (30.9)	1.00	1.00
Disclose HIV status to partner n = 223				
Yes	90 (40.4)	105 (47.1)	3.14 (1.22, 8.09)**	4.09 (1.26, 13.30)**
No	6 (2.7)	22 (9.9)	1.00	1.00
Partner HIV status (n =203)				
Positive (concordant)	74 (36.5)	69 (34.0)	1.00	1.00
Negative (discordant)	20 (9.9)	40 (19.7)	2.15 (1.14, 4.02)**	2.05 (1.012, 4.15)**

Note: ***p < 0.001, **p < 0.05.

PLHIVs marital length less than 4 years 5.5 times [AOR, (95% CI), 5.5(2.08-14.51), from 5-9 years 4.8 times (2.14-10.78) and 10–14 years were 2.8 time (1.20 - 6.63)] more likely to have fertility desire as compared to had more than 15 years marital length. The majority of in-depth interview discussants supported this finding, for instance: as one 30 years female discussant stated: “I want to give birth because my husband strongly desire to have children to replace ourselves, so I have to get pregnant after one year. I stayed with my husband for 6 six years without having a child and marriage without children is meaningless and does not long last. PLHIVs who had no biological living child was 11.4 times [AOR, (95% CI), 11.42(3.27- 39.90) and 1 to 3 children were 3.67 times (1.27- 10.62)] more likely to have fertility desire as compared to had more than 4 biological living children. PLHIVs who faced community pressure for child birth was 3.67 times [AOR, (95% CI), 3.67(1.54 -8.70)] more likely to have fertility desire as compared to not faced community pressure for child birth. Those who have partner’s had fertility desire was 7.18 times [AOR, (95% CI), 7.18(3.39- 15.22)] more likely to have fertility desire as compared to those who have no fertility desire partner.

Among HIV related treatments and social support variables those enrolled for ART less than or equal to ≤1 years was 5 times [AOR, (95% CI), 5.0(1.91- 13.09)] more likely to have fertility desire as compared to more than 5 year since enrolled to ART and also the majority of in-depth interviews discussants supported this, for example:-as one 40 years Male discussant explained:” I need a child strongly. It gives me self-esteem and value so, no loneliness. I proved PLHIVs can get negative child that is why I desire strongly after five years. I have a three and half year old negative child which I got after started ART and happy now”.

PLHIV disclosed HIV-serostatus to their partner was 3.9 times [AOR, (95% CI), 3.9(1.37- 11.10)] more likely to have fertility desire as compared to those not disclosed their serostatus to their partner. PLHIVs partner discordant was 2 times [AOR, (95% CI), 2.05(1.01- 4.15)] more likely have fertility desire as compared to those concordant HIV-serostatus partner (Table 4).

## Discussions

The prevalence of fertility desire of PLHIV in this study was 39.1%, which is lower than EDHS 2011, study conducted in Cape Town South Africa and Nigeria, which account 63%, 51% and 63.3% respectively [3,13,17]. This may be due to different sociodemographic characteristics of the populations and cultural difference towards having large family size and fertility rate.

In this study PLHIV who mentioned their main reasons for current fertility desire were wanted at least one child to replaced themselves, did not have desired number of children and believed that by using ART/PMTCT to get HIV free baby (perceived efficacy of PMTCT and ART) and others reasons like to strength their marriage and replacing previously died baby. This is similar with study done in Rural Malawi rural, Uganda, New Guinea, Ethiopia (Addis Ababa and Nekemte) in which their main reasons were: wanting at least one child/more children, to reach ideal family size, no live child, wanting a different sex, support in old age, family/partners influence to have children, marriage, replacing previous died child and sufficient financial means as important in their fertility desires for PLHIV [15,16,18-20]. These indicate that the need for reproductive health services of PLHIVs in HIV care settings were need more comprehensive care in order to meet the PLHIV’s diverse reproductive intentions for those who are intended to have children.

Multivariable logistic regression indicates that age was significant predictors of fertility desire with age within 18–29 and 30–39 years were 4 and 3.9 times more likely have fertility desire as compared to age 40 years respectively. This is similar with studies in Brazil, United States, South Africa, Uganda and Nigeria [12,21-24] have showed that younger PLHIV are more likely to desire (more) children than older PLHIV. The possible explanation may be due to that relatively older PLHIV have already achieved, or are closer to achieving, their desired family size than younger PLHIV. This has public health importance as many new HIV infections are occurring in younger PLHIV.

The number of surviving children was predictor of fertility desires PLHIV who have no biological living children and those who have few or 1 to 3 children were 11.4 times and 3.6 times more chance to have fertility desire as compared to those who have more than 4 biological living children. This finding is in line with other studies done in the United States, Brazil; Nigeria and Malawi and in Ethiopia studies in Addis Ababa and Nekemte town [15,16,20,21,24,25] and South Africa [13,22], Uganda [18,23]. PLHIV, like anyone else, continue to desire (more) children until they achieve their desired family size.

PWLHAs marital length less than 4 years, within 5-9 years and 10–14 years were 5.5, 4.8 and 2.8 times were more likely have to fertility desire as compared to those marital lengths more than 15 years. This may be due to relatively PLHIV who have longer marital duration have already achieved, or are closer to achieving, their desired family size than shorter marital length PLHIVs’.

PLHIV’ faced community pressure for having children was 3.7 times more likely have fertility desire as compared to those not faced community pressure for having children. This finding is consistent with studies in many societies and especially in SSA with those who are childless receiving negative social disapproval. The value of children in the identity and social status of men and women applies to PLHIV as well, and so they are under intense pressure from family, spouses and friends to reproduce [5,26,27]. This may be due to having children may re-establish the PLHIV with higher self-esteem, may help them restore a sense of normalcy in family life and health, and alleviate (mitigate) the potentially dehumanizing effects of living with HIV. Partner fertility desire was 7 times more likely have fertility desire as compared to those who have no partner. This is in line with the study done in South Africa and Ethiopia in Addis Ababa, South Wollo and Nekemte town [16,20,22,28]. This suggests that family planning and fertility related issues information should focus on partner as well.

PLHIV duration since HIV-diagnosis less than or equal to ≤1 years was 5 more likely to have fertility desire as compared to those who more than 5 year. One explanation would be that the PLHIV enrolled to ART for a longer period might have gone through extensive health education that might have influenced their intentions unlike those that have just enrolled to ART. The influence of longer time since diagnosis of infection probably reflects the cumulative effects of decisions made by individuals who had weighed the consequences of their wish for parenthood over several months or years. Health professionals at different levels of institution should to be discontinuing from the conventional systematic advice against pregnancy but, in addition to laying emphasis on the risks, provide adequate information on the efficacy of PMTCT and practicable reproductive options for HIV-positive individuals.

PLHIV disclosure HIV-serostatus to partner was 4 times more likely have fertility desire as compared to those who do not disclosed their serostatus to their partner. This may be due to those who had disclosed their status have more discussion on their desired number of children than others and also PLHIV may want to have children to avoid stigma and secrete their status; to ensure family continuity in the future, to have offspring of their own to perpetuate their name and lineage after they die, and to be supported in old age.

PLHIV partners serodifference or discordant was 2 times more likely to have fertility desire. This is in line with other studies conducted in Uganda and Burkina Faso [23,29]. This observation could be explained by the fact that, as this study shows it, some young people still ignore the modes of HIV infection. Moreover, the issue of conception would be particularly important as means to avoid partner infection should be vital that needs consideration on alternative options/technologies. Health professional’s work in ART clinics should be given attention for those coming for VCT and Provider Initiated HIV test and counseling by providing health messages about fertility, vertical transmission of HIV for discordant couples in this context is vital to ensuring informed reproductive decisions among PLHIVs and also encourage on disclosure of HIV-serostatus to their partner. On this research by considering the main strength of this research lies in its computer generated random sampling strategy for data collection, and the fact that used qualitative method to supplement the result and also to explore factors that are not addressed by quantitative survey. A set of reliability and validation rules were applied and all associated factors were taken after indication of significance in the “goodness of fit” for the models. Even though this study also had a few limitations: This study was facility-based among PLHIVs’ that results were not generalizable to the general population in the community and cause and effect relation was not assured because of cross-section study deign.

## Conclusion

The prevalence of fertility desire of PLHIV in the study area was 39.1%. In this study:- age, marital length, biological child, partner, community pressure, duration of HIV-diagnosis, discordant HIV-test and disclosure of HIV-serostatus to partner were demonstrated to have more fertility desire among PLHIV and these factors should be emphatically considered during PLHIV’s reproductive health program development. Therefore, Policy makers and health planners in the developing countries of sub Sahara African would better to plan and adapted assisted reproductive option/technologies for discordant partner which contribute to decrease HIV- new infections to sexual partner and new born.

## Abbreviations

AIDS: Acquired immunodeficiency syndrome
ART: Antiretroviral therapy
AOR: Adjusted odd ratio
COR: Crude odd ratio
HAART: Highly active antiretroviral therapy
EDHS: Ethiopian demographic and health survey
FHAPCO: Federal HIV/AIDS prevention and control office
FMOH: Federal Ministry of Health
HIV: Human immunodeficiency virus
JU: Jimma University
MTCT: Mother-to-child transmission
PMTCT: Prevention of mother to child transmission
PLHIV: People living with HIV
SPSS: Statistical Package for Social Sciences
SSA: Sub Saran African
UNAIDS: Joint United Nations Programme on HV/AIDS
USD: USA dollar
WHO: World Health Organization
Y: Years
